# Tailored Limonene-Based Nanosized Microemulsion: Formulation, Physicochemical Characterization and *In Vivo* Skin Irritation Assessment

**DOI:** 10.34172/apb.2021.040

**Published:** 2020-08-09

**Authors:** Mohammed M Mehanna, Kawthar Khalil Abla, Hoda A. Elmaradny

**Affiliations:** Department of Pharmaceutical Technology, Faculty of Pharmacy, Beirut Arab University, Beirut, Lebanon.

**Keywords:** D-limonene, Essential oil, Gelucire® 44/14, Solid wax, Microemulsion, Pseudo-ternary phase diagram, Skin irritation

## Abstract

***Purpose:*** Microemulsion (ME) achieved progressing consequences on both the research and industry levels due to their distinctive properties. ME based-limonene system is considered as a surrogate to the traditional microemulsion composed of conventional oils. Thus, a novel microemulsion based on D-limonene and Gelucire® 44/12 had been designed and evaluated with assessing the factors affecting its physicochemical characteristics and in vivo skin irritation.

***Methods:*** The impact of microemulsion components and ratios on the isotropic region of the pseudo-ternary phase diagram was investigated. The optimal formula was evaluated in terms of percentage transmittance, average globule size, size distribution, zeta potential, microscopical morphology, stability under different storage conditions and its effect on the mice ear skin.

***Results:*** The results demonstrated that Labrasol® and Labrafil® M 1944 CS had been selected as surfactant and co-surfactant, respectively, due to their emulsifying abilities. The largest isotropic area in the pseudo-ternary phase diagram was at a weight ratio of 4:1 for Labrasol® and Labrafil® M 1944 CS. The optimized microemulsion with 25% w/w of the lipid phase and 58.3% w/w of the aqueous phase displayed an optical transparency of 96.5±0.88 %, average globule size of 125±0.123 nm, polydispersity index of 0.272±0.009, zeta potential of -18.9± 2.79 mV with rounded globules morphology and high stability. The in vivo skin irritation and the histopathological evaluation of microemulsion elucidated its safety profile when applied on the skin.

***Conclusion:*** The formulated microemulsion is a prospective aid for an essential oil to minimize its volatility, enhance its stability, and mask its dermal irritant.

## Introduction

Phytomedicine refers to the use of herbs and plants to relieve and alleviate human disorders. It has become an important aspect of flourishing worldwide commercial health scheme.^1^ Essential oils or aetherolea are intense hydrophobic fresh liquids, containing volatile chemical compounds that are extracted from plants.^2^ These oils consist mainly of terpene and other oxygenated compounds like phenols and alcohols. They can exert a vast array of therapeutical and biological effects, such as antibacterial, antifungal, anti-inflammatory, antiviral, anticancer, and many others.^3^ Recently, many studies have focused on the significance of these natural compounds and their outcomes on human health. Tea tree oil, for instance, was able to inhibit biofilm formation of *Streptococcus pyogenes, Pseudomonas aeruginosa, Staphylococcus aureus,* and *Proteus vulgaris* as as revealed by Kabir and Mahboob in their investigation.^4^ Moreover, thyme oil clinically reduced the gene expression and the production of pro-inflammatory mediators interleukins 1-beta and interleukins 6, and increased interleukins 10 cytokines in macrophage THP-1 cells in acute monocytic leukemia patients.^5^

**Figure 1:**
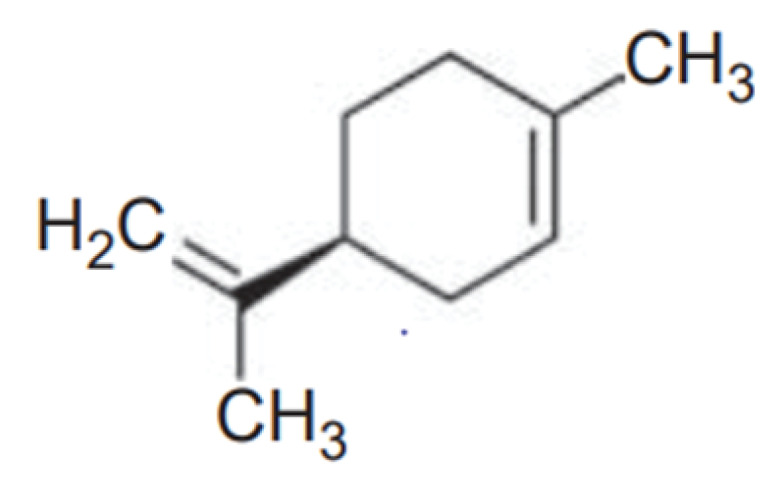
(R)- (+)-Limonene.

Essential oils can be divided into three structural classes based on their toxicological potential. Limonene can be classified as class one due to its high safety profile and low oral toxicity.^6^ D-limonene (1-methyl-4-isopropyl-cyclohexene) is a member of the terpene family, with an aliphatic hydrocarbon structure, and molecular formula C_10_H_16_ as shown in Figure 1.^7^ Its main source is the rind of citrus fruits like lime, mandarin, orange, and grapefruit. It is optically active and found as two enantiomers; R (D-limonene) which more commonly occurs and S (L-limonene) which is found in mint.^8^ Limonene is a relatively unstable monoterpene as it degrades into isoprene upon increasing the temperature, and it oxidizes under the effect of high humidity. Thus, proper storage of limonene, such as the suitable type of glass, the temperature, and the light, can drastically prolong its shelf life and improve its quality as well as its pharmacological action.^9^ Lately, numerous therapeutic and pharmacological effects of D-limonene have been studied broadly in many investigations. For example, Jing et al^10^ had reported that limonene oil provided a reduction in serum triglyceride, low-density lipoprotein, glucose tolerance, and fasting blood glucose level and an increase in high-density lipoprotein. Also, de Souza et al^11^ proved that limonene had gastro-protective activity by increasing the mucus production of the stomach as well as modulating the oxidative stress, leading to local mucosal protection. Despite the benefits of essential oils, the most common side effects that should be taken into account are skin irritation and contact dermatitis upon their topical application.^12^

Microemulsion (ME) is a dynamic structure, isotropic, clear, and nano-dispersive colloidal system consisting of microdomains lipid(s) (a mixture of olefins and various hydrocarbons) and water. MEs can be stabilized by interfacial nonionic surfactants and in most of cases co-surfactants.^13^ MEs are thermodynamic stable dispersion with a droplet size less than 150 nm.^14^ This system has gained attention for many reasons chiefly; the ease of scaling-up and manufacturing, nanometer-sized particle with minimal aggregation, elegant and clear appearance, ability to enhance lymphatic transport and dissolution rate of lipophilic drugs, which give an edge over other delivery systems like dendrimers, liposomes, and polymeric nanoparticles.^15^ The applications of MEs are span and plenteous in many areas including cosmetics, chemical reactors, biotechnologies, and drug delivery. Liu et al^16^ had designed a curcumin-loaded micoremulsion system composed of limonene as an internal phase. Their results showed a significant increase in its transdermal delivery, by reducing the viscosity and the diffusional barriers of the stratum corneum that act as an obstacle for the transdermal route. Also, cinnamon oil with its antimicrobial activity had been used as the lipid core in the microemulsion system and showed a significant bacterial inhibitory activity compared to cinnamon oil-free preparations (*P* < 0.05) could disrupt the rod shape of *E. coli* with a non-specific mechanism.^17^

Many current studies have highlighted the importance of essential oils in microemulsions as a vehicle for different therapeutic agents.^18-20^ Yet, limited numbers of researches investigated the efficiency of solid lipid microemulsion in combination with terpene oil to form a stable nano-dispersive system. In the current study, Gelucire^®^ 44/14 has been selected as solid lipid due to its low melting point (43°C), self–emulsifying properties, its excellent surfactant property which enhances the wettability of active pharmaceutical agents *in vitro* and *in vivo*, its ability to form fine emulsion upon contact with the aqueous fluid and its ability to develop a sustained release formula.^21^

The study aimed to formulate a skin-friendly ME system composed of Gelucire^®^ and limonene by spontaneous emulsification method. The selection of the formula was carried out according to the pseudo-ternary phase diagram. The selected formula was optimized by assessing its average droplet size, polydispersity index (PDI), and zeta potential. Transmission electron microscope (TEM) was used to evaluate the morphology of the formed nano-droplets, in addition to its stability which was also evaluated under different conditions for three months. *In vivo* skin irritationtest was also carried out to detect the effect of pure limonene oil and limonene-based ME preparation on mice ears for seven consecutive days.

## Materials and Methods

### Materials 

(R)-(+)-Limonene, propylene glycol, polyethylene glycol 400 (PEG 400), and PEG-40 hydrogenated castor oil (Cremophor^®^ RH 40) were purchased from Sigma Co. (Sigma-Aldrich, Steinheim, Switzerland). PEG-8 Caprylocaproyl polyoxyl-8 glycerides NF (Labrasol^®^), oleoyl polyoxyl-6 glycerides (Labrafil^®^ M 1944 CS), and lauroyl polyoxyl-32 glycerides (Gelucire^®^ 44/14) were kindly donated by Gattefosse Co. (Lyon, France). All other chemicals and solvents used were of analytical grade.

### Miscibility of Gelucire^®^ and D-limonene oil 

Gelucire^®^ was mixed with limonene in five different ratios (5:1, 3:1, 1:1, 1:3, 1:5) to evaluate their miscibility in the preparation. The lipid mixtures were heated at 47±2°C and mixed at a rate of 600 rpm on a magnetic stirrer for 15 minutes. The mixtures were then allowed to be cooled at 4±2°C before checking for any sign of phase separation or turbidity that may occur by visual inspection.^22^ Miscibility was also evaluated by diluting one ml of the prepared mixtures with 100 mL of deionized water and checked for the existence of unimodal size distribution by Zeta sizer 2000 (Malvern Instrument, UK) at 25±2°C and at 90°C scattering angle.

### Preliminary screening of surfactants 

The selection of the suitable surfactant was based on the measurement of percentage transmittance and ease of emulsification according to the method described by Shah et al.^23^ with some modifications. Briefly, 300 mg of each surfactant was added to 300 mg of the lipid phase (Gelucire^®^ and limonene). The mixture was heated at 47±2°C and mixed on a magnetic stirrer at 600 rpm till complete homogenization. Fifty mg of the isotropic mixture was then diluted with deionized water to 100 m» in a stopped conical flask. The emulsification was assessed according to the numbers of flask inversions required to produce a uniform dispersion emulsion after two hours, and % transmittance was measured at 638.2 nm (Jasco V-730 spectrophotometer). The surfactant that was able to form a dispersion with the highest percent transmittance was chosen.

### Preliminary screening of co-surfactants 

The selected surfactant and the lipid phase were used to evaluate the effectiveness of the co-surfactants to improve the emulsification capability of the system. The selected surfactant was mixed individually with each of co-surfactants and added to 300 mg of the lipid phase. The mixture was mixed at 47±2°C on a magnetic stirrer at 600 rpm till complete homogenization. Then, 50 mg from each mixture was diluted with deionized water to 100 m» in a conical flask and kept for 2 hours. Each dispersion was evaluated as previously described in section 2.3.

### Construction of pseudo-ternary phase diagram 

Pseudo-ternary phase diagram of lipids, surfactant/co-surfactant mixture (Smix), and deionized water were constructed using spontaneous emulsification method with the help of CHEMIX school 3.51 software version 7 (Arne Standnes, Bergen, Norway). It was constructed to obtain the concentration range of the selected components that were able to form the largest monophasic ME region on the diagram. Different constant weight ratios between Labrasol^®^ and Labrafil^®^ (km) of Smix were prepared (1:3, 1:1, 2:1, 3:1, 4:1, 5:1) then mixed with the lipid phase to give the weight ratios (w/w) of 9:1, 8:2, 7:3, 6:4, 5:5, 4:6, 3:7, 2:8, and 1:9. Deionized water was then added dropwise to each mixture under vigorous stirring (600 rpm) using a magnetic stirrer until the mixture became transparent or nearly transparent. The concentrations of the used components were marked by points on the phase diagram and the resulting area which is covered by these points identified the microemulsion region.^24^

### Preparation of limonene-based microemulsion 

The preparations were simply performed by mixing the lipids at the selected ratio on 47±2°C for 15 minutes using a magnetic stirrer at a rate of 600 rpm, followed by the addition of the selected surfactants and co-surfactants at the same temperature. Deionized water was then added dropwise until a clear dispersion was formed.^25^

### Physicochemical characterization of limonene-based microemulsion 

#### Macroscopical examination 

Transparency, color, homogeneity, and phase separation of the freshly prepared microemulsions were checked.

#### Percentage transmittance 

The percentage transmittance of the prepared microemulsion was measured at 638.2 nm using deionized water as a blank.^26^

#### Globule size analysis 

Globule size and PDI of the formulated microemulsions were measured using zeta sizer 2000 at 25±2°C and at 90 degrees scattering angle. To avert the multi-scattering phenomena, each prepared sample was diluted with deionized water before the analysis.^27^

#### Zeta potential measurement 

Zeta potential analysis of the prepared ME was assessed using Malvern Zetasizer. Samples were diluted with deionized water before each measuring. One milliliter of the diluted samples was placed in a folded capillary cells supported with platinum electrodes. The values were calculated using the Dispersion Technology software built into the Malvern Zetasizer.^28^

#### pH measurement 

The pH of the optimized ME was examined by directly immersing the pH electrode into the sample using a calibrated digital pH meter (Model: SED 12500 V, Martini Instruments Co., Ltd., Beiging) at room temperature (25±0.2℃).^29^

#### Refractive index 

The refractive index of the formulated microemulsion was measured by placing one drop of the system on the slide of the digital refractometer (Model REF 123, China).^30^

#### Transmission electron microscope (TEM) 

Structural and morphological elucidation, as well as the size of the optimized microemulsion were observed by TEM (JOEl JEM CX 100 electron microscope). The prepared microemulsion was diluted with deionized water in a ratio 1:1000, then a drop from the diluted dispersion was placed on a copper grid and the excess sample was removed with a filter paper. The sample was stained with uranyl acetate solution and the grid was dried at room temperature before examination.^31^

#### Robustness of microemulsion 

The formed microemulsion was diluted in two different ratios (1:10 and 1:100) with deionized water. The diluted MEs were stored for 24 hours at room temperature (25±0.2℃) to evaluate dilution stability and assess for any signs of phase separation.

#### Thermodynamic stability 

The prepared ME was diluted to 100 mL with deionized water, the diluted samples were then exposed to centrifugation at 3000 rpm for 30 minutes and examined for any change in phase behavior. Additionally, ME was subjected to three completed freeze-thawing cycles consisting of 24 h at -5±2℃ followed by 24 hours at 25±2℃ to evaluate their stability under stressful conditions.^32^

### In vivo skin sensitivity assessment 

#### Animals 

Irritation test and histopathological examination of the free and incorporated limonene oil in ME system was carried on male BALB/c mice weighing between 20-25 g. Animals were procured by the animal house of the Faculty of Pharmacy of Beirut Arab University, Beirut, Lebanon. The mice were housed in polyacrylic cages under standard laboratory conditions with dark-light cycles (12/12 h) before and during the experiments. Mice had a standard chow diet and free access to water.

#### Skin irritation test 

To assess and compare the effect of pure limonene oil and limonene-based microemulsion system, the study was carried on mice. The animals were divided into two groups each of three. One hundred milligram of each of pure limonene oil and limonene-based ME was applied on the right ears of the mice with a uniform spreading, keeping the left ears as control. The oil and the prepared formula were removed after 24 hours and the ears were checked for any development of edema or erythema.^33^ To investigate the accumulative result of repeated application, the oil and the prepared ME were applied again on the same area, once per day for the next 7 days. Draize scale was used to assess the skin irritation after 24 hours and after 7 days. The results were graded from 0 to 4, where 0 indicates no erythema and no edema, 1 indicates very slightly erythema or edema, 2 indicates well-defined erythema or slight edema, 3 indicates moderate to severe erythema or slight edema and 4 refer to severe erythema (beet redness) to slight eschar in formations (injuries in-depth) or severe edema.^34^ This was carried out by visual scoring as previously reported by Guan et al.^35^

#### Histopathological examination 

The effect of pure limonene oil and limonene-based microemulsion system on the skin of mice after consecutive once daily application for 7 days was studied. The remaining oil and formula were removed gently using a cotton swab, which was already dipped in 0.9% w/v physiological saline solution. Thereafter, mice were sacrificed and the excised skin was collected and maintained in a 10% formalin solution. Tissues were dehydrated with ethanol and firmed in paraffin wax. The skin samples were then stained with eosin and hematoxylin (E&H) and cut into 3-4 μm with a rotary microtome (cut 5040, Micrototec, Walldorf) to be examined under a light microscope to check if any histological irregularity had occurred in the underlying tissues after applying pure limonene oil and the ME formulation.^36^

#### Short-term stability study 

Storage stability of the prepared limonene-based microemulsion was studied at different temperatures, 25, 5, and 0±2°C, for 3 months and evaluated for any sign of precipitation or separation. The stored dispersions were also assessed for average particle size, PDI and zeta potential every 4 weeks.^37^

### Statistical analysis

The obtained results from various tests were expressed as mean (n = 3). The standard deviation (mean ± SD) was analyzed by a *t* test or one-way analysis of variance (ANOVA) test. *P*value<0.05 considered to be significant using GraphPad Prism version 8.0.2 (GraphPad Software, San Diego, CA).

## Results and Discussion

### Miscibility of Gelucire^®^ and D-limonene oil 

The spatial distribution and the miscibility of lipids, oils and other excipients in the nano-formulations can impact the release rate of the drug and reproducibility of the process.^38^ Thus, the miscibility between Gelucire^®^ and limonene was initially evaluated by visual inspection (macroscopical appearance) upon heating and cooling the prepared formulas with different weight ratios. Visual inspection allowed to assess the presence of oil droplets between the lipid and the oil or on the surface of the preparations. Macroscopic appearance can also help in figuring out any sign of phase separation phenomena or turbidity induced by the cooling of the melted mixtures.^22^ When an equal amount of Gelucire^®^ and limonene were mixed and melted together (1:1), it formed a clear, transparent and homogenous phase mixture, while the other ratios showed less clear to turbid appearance (Table 1).

**Table 1:** Macroscopical appearance and polydispersity index (PDI) of different weight ratios of limonene and Gelucire^®^ mixtures

**Ratio of limonene to** **Gelucire** ^®^ **(w/w)**	**Appearance upon cooling**	**Polydispersity index** ^a^	**Number of peaks** ^b^
5:1	Turbid	0.652±0.451	3
3:1	Less clear	0.449±0.115	2
1:1	Clear	0.299±0.032	1
1:3	Less clear	0.334±0.299	2
1:5	Less clear	0.308±o.410	2

^a^Mean ± SD (n = 3). ^b^Appeared in the particle size distribution graph (unimodal, bimodal, and multimodal).

Nevertheless, despite of these mixtures are being macroscopically miscible, they may have microscopic heterogeneities that can result in poor stability and phase separation during storage.^38^ Subsequently, for additional confirmation, the PDI of the mixtures was measured and evaluated, where small PDI values reflect a unimodal particle size distribution while higher values mean that the distribution is multimodal. The weight ratio (1:1) was able to form nano-droplets with the smallest PDI (0.299±0.032) with a single peak as shown in Table 1, which indicated full miscibility and a monodisperse system formation with a uniform distribution of Gelucire^®^ and limonene.

### Selection of surfactant and co-surfactant 

The most critical limitation related to the microemulsion or nanoemulsion-based systems is the toxicity of the utilized components. A large concentration of surfactants may result in gastrointestinal problems when administrated orally or skin irritation when applied topically. Thus, the cautious selection of surfactants, as well as co-surfactant, became necessary.^39^ Further, the proper selection of surfactant and co-surfactant in the ME systems, is very crucial since they are adsorbed at the interface, emulsify the lipid system spontaneously, decrease the interfacial energy and provide a mechanical defy to the coalescence.^40^ In this context, the selection of surfactant and co-surfactant was based on measuring and comparing their percentage transmittance which reflect their ability to emulsify the lipid phase without any input energy.^23^

**Figure 2:**
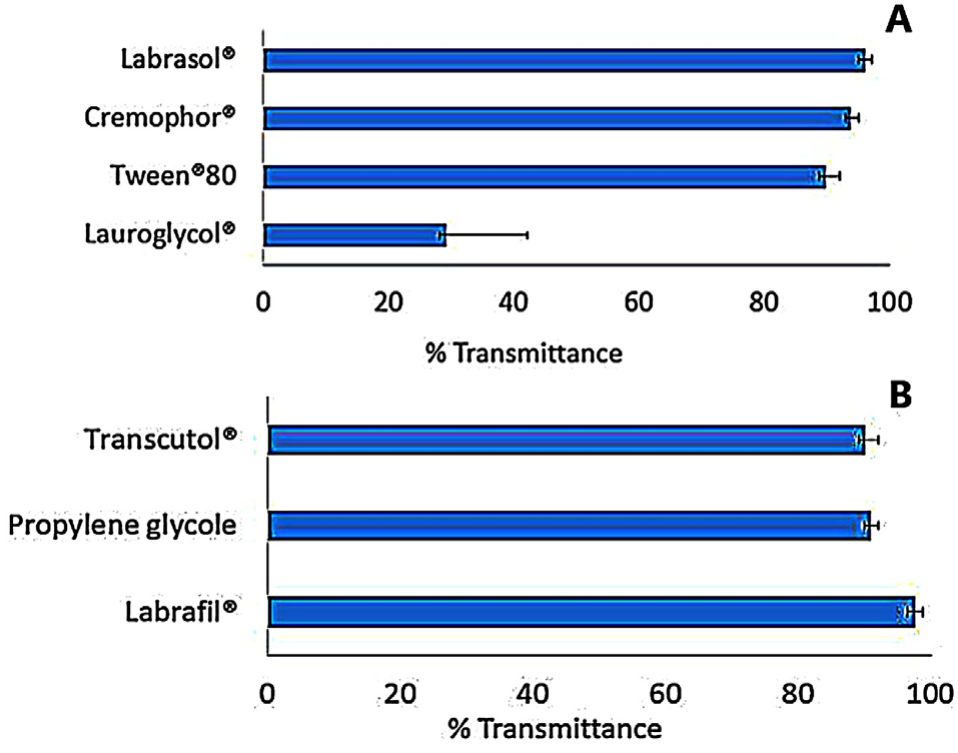
Emulsification efficiency of (A) various surfactants (B) and co-surfactants of limonene and Gelucire® mixtures.

As shown in Figure 2A, Labrasol^®^ had a very good ability to emulsify as it required only three flask inversions for clear emulsion formation with the highest percentage transmittance (96.2±0.122%), followed by Cremophor^®^ RH 40 and Tween^®^ 80 that required the same number of flask inversions with percentages transmittance of 93.9±0.52% and 89.9±0.135%, respectively. This result was in line with the study performed by Kumar et al^41^ who revealed that microemulsion prepared with Labrasol^®^ resulted in the formation of a microemulsion system with a high percentage transmittance value of 99.87%. Labrasol^®^, a non-ionic surfactant, is a PEG derivative of medium-chain fatty acid triglyceride chemical structure. It is one of the best surfactants used since it has a good solubilization property, low toxicity profile and low critical micelle concentration.^42^ Moreover, its HLB value is high and equal to that of Gelucire^®^ (both of HLB = 14) resulting in excellent microemulsification feature (high % transmittance) and maximum stability of the system.^43,44^ On the other hand, Lauroglycol^®^ revealed low emulsification property since it required eight flask inversions to give a clear emulsion with lowest percentage transmittance 29.2±0.221% (Figure 2A). This may be due to the presence of co acid backbone, which has a long chain in its structure (large molecular volume) that reduce its emulsification power upon incorporation in microemulsion.^45^

The selection of the co-surfactant is a critical step due to its ability to decrease the surfactant concentration, providing more stable microemulsion, improving polydispersibility of the system, as well as ensuring the flexibility of the interfacial surfactant film.^46^ In the present investigation, four co-surfactants, namely; Labrafil^®^, Transcutol^®^, propylene glycol and PEG 400, were examined. Polyethylene glycol was excluded from the evaluation due to its inability to form an emulsion upon mixing with the lipid phase and Labrasol^®^. As portrayed in Figure 2B, the addition of Labrafil^®^ to the microemulsion, induced very good emulsification with a high percentage transmittance (97.6±0.551%), followed by Transcutol^®^ with a percentage transmittance 91±0.336%, to end with propylene glycol (90.2±0.354%) (*P*< 0.05). From the previous results, Labrafil^®^ was selected for further study as co-surfactant.

### Pseudo-phase diagram of limonene-based microemulsions 

**Figure 3:**
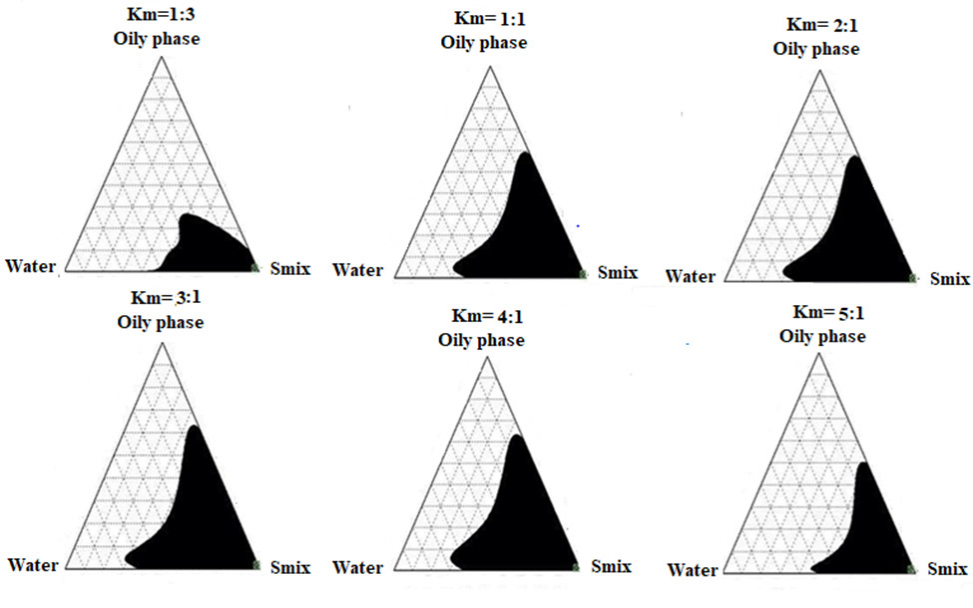
The pseuodo-ternary phase diagrams. Shaded areas corresponding to monophasic transparent microemulsion at different km values (Labrasol^®^ to Labrafil^®^) with different aqueous to oily phases (Gelucire^®^ and limonene).

To obtain the appropriate concentration ranges of the selected components for ME formula, the pseudo-ternary phase diagram was designed for six Smix ratios (1:1, 2:1, 3:1, 4:1, 5:1, 1:3) to locate the optimal formula composition. The six ratios of Smix were able to form clear and stable microemulsions, yet the constant weight ratio between Labrasol^®^ and Labrafil^®^ (km) which resulted in the largest isotropic region in the phase diagram was found to be 4:1, and thus was selected for further studies. These results are clearly illustrated in Figure 3, and by using Sketch and Calc^®^ program that calculated and confirmed the largest microemulsion area for 4:1 w/w ratio. The analysis diagram revealed that increasing the weight ratio of Labrasol^®^ from 1% to 4% (w/w), led to an expansion of the microemulsion region. This result was in agreement with Patel et al^26^ in which 4:1 Smix analysis diagram of the prepared microemulsion included the largest isotropic area. That could be explained based on the fact that further depletion of the interfacial tension, increasing in the fluidity of the interface, and enhancement of the entropy of the microemulsion system occurred by increasing the surfactant concentration.^39^ Yet, it was noted that increasing the weight ratio of Labrsaol^®^ from 4% to 5% (w/w), did not cause any significant enhancement in ME region and this was due to the presence of Labrafil^®^, that acts mainly in reducing the concentration of Labrasol^®^ required to emulsify the mixture, as well as increasing fluidity of the interface. Hence, the effect of Labrasol^®^ relative concentration on the microemulsion area greatly depends on the presence and concentration of Labrafil^®^ which solely cannot achieve a sufficient reduction of oil/water interfacial tension, and thus unstable microemulsion can be formed.^47^

After the selection of the optimal Smix weight ratio (km = 4:1), nine different ratios (9:1 to 1:9) between the oil phase and aqueous phase were prepared and evaluated for their visual appearance, droplet size, and PDI. As shown in Table 2, F7 which consisted of 25% of oil phase (Gelucire^®^ and limonene), 58.3% w/w of Smix (46.64% Labrasol^®^ and 11.66% Labrafil^®^), and 16.7 % w/w of deionized water, showed a bluish transparent appearance, with small droplet size (125 nm ± 0.12) and the smallest PDI value of 0.272 ± 0.009.

**Table 2:** Composition of limonene-based microemulsion formulations constructing the pseudo-phase diagram (km=4:1)

**Formula**	**Components**			**Appearance**	**Size (nm)** ^a^	**PDI values** ^a^
	**Limonene & Gelucire** ^®^ **(%w/w)**	**Labrasol** ^®^ **& Labrafil** ^®^ **(%w/w)**	**Deionized water** **(%w/w)**			
F1	85.71	9.52	4.76	Turbid	278.2±23.43	0.699±0.193
F2	76.19	19.04	4.76	Turbid	292.3±33.80	0.72±0.220
F3	64.22	27.52	8.25	Turbid	201.1±19.62	0.695±0.201
F4	50.84	33.9	15.25	Slight turbid	178.5±12.41	0.671±0.184
F5	43.10	43.10	14.53	Slight turbid	177.6±9.80	0.485±0.121
F6	34.18	51.3	14.53	Bluish transparent	153.2±3.20	0.42±0.052
F7	25	58.3	16.7	Bluish transparent	125±0.12	0.272±0.009
F8	16.8	67.23	15.97	Bluish transparent	120.4±2.32	0.483±0.050
F9	8.54	76.92	14.53	Slight turbid	145.1±14.60	0.311±0.080

^a^ Mean ± SD (n= 3).

### Physicochemical characterization of the optimized microemulsion 

#### Particle size analysis and PDI 

The particle size, PDI, and % transmittance are promoting requirements for the biopharmaceutical execution of the microemulsion system. Table 2 illustrated that F7 was homogeneous monophasic ME having a bluish transparent appearance at room temperature (25±2°C), with a small particle size less than 150 nm (125±0.12 nm) which met the acceptance criteria as reported by Bshara et al.^48^ The small droplet average size of microemulsion resulted from the presence of relatively high concentrations of the surfactant (Labrasol^®^) and co-surfactant (Labrafil^®^) in the system which led to increasing the microemulsion system entropy, lowering the oil/water interfacial tension and decreasing its free energy.^28^ Moreover, the small PDI (0.272±0.009) reflects a unimodal particle size distribution with a minimal proneness to agglomeration.^28^ High percentage transmittance after diluting the optimized microemulsion with 100 ml deionized water was 96.5±0.88%, revealing efficient emulsification of Gelucire^®^ and limonene by Labrasol^®^ and Labrafil^®^.

#### Zeta potential 

Surface zeta potential is an important parameter since it reflects the degree of repulsion between adjacent nano-droplets whether are negative or positive charged nanoparticles^49^ and can predict the physical stability of the nanosized ME system, where zeta potential value of ±30 mV can award a thick high energy diffusion barrier that prevents the flocculation or coagulation of the nanoparticles achieving good colloidal stability.^50^ In this study, the system showed a quit low zeta potential (-18.9±2.79 mV), which was less than ≤ 30 mV; yet, the high amount of non-ionic surfactant (Labrasol^®^) may create a steric stabilization that provides a rigid film surrounding the oil droplets and prevents self-coalescence.^28^ This result is linked to the use of non-ionic surfactant which did not induce any charge on the ME surface. A similar result was observed by Moghimipour et al^29^ where the use of non-ionic surfactants (Tween^®^ 80 and Span^®^ 80) during fabricating ME systems resulted in relatively low zeta potential values ranged from 0.84 mV to -18 mV. On the other hand, Aloisio et al^51^ designed anionic MEs using sodium oleate, where the ME surface charges were of high zeta potential values (between -52 and -66.1 mV). These observations confirmed the effect of surfactant nature on the overall charge of the ME system.

#### pH and refractive index 

The pH value of formula (F7) was slightly acidic (6.2±0.3) and this could be interpreted through the presence of Labrasol^®^ as a surfactant with high concentration (46.8 %). Labrasol^®^ structure has fatty acid triglyceride of caprylic and capric acid which decrease the pH value of the formula, yet this slight acidic value is still within the required physiologic pH range accepted for different dosage forms. This finding is incoherent with the ME system which prepared by Okur et al^52^ that Labrasol^®^ as a surfactant displayed a pH value of 6.0±0.01.

The refractive index is the net value of the components of microemulsion and indicates the isotropic nature of the formulation.^30^ The refractive index of F7 was 1.43 which is close to that of water and limonene (1.33 and 1.47 respectively), revealing a good homogeneity (isotropy) of the ME formulation.

#### Transmission electron microscope 

**Figure 4:**
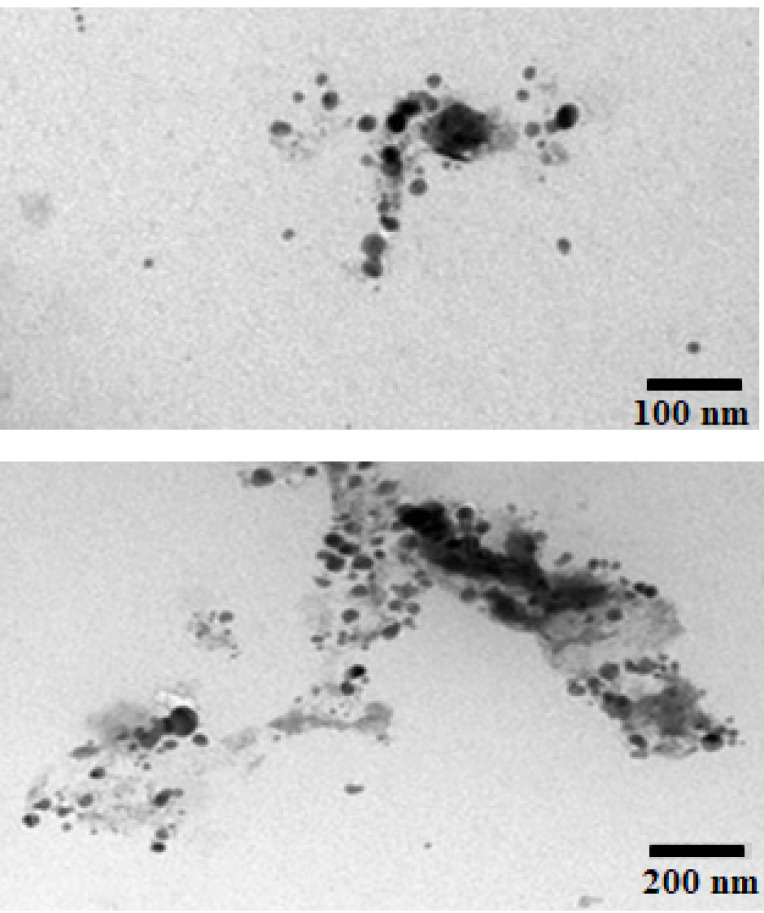
Transmission electron microscopical images of the optimized limonene-based microemulsion (F7).

Structural and morphological elucidation of the limonene-based microemulsion system (F7) was analyzed using TEM as showed in Figure 4. The micrographs revealed relatively separate ME droplets with smooth spherical outlines. The hydrodynamic diameter of most of the droplets in the image was in the range of 100–200nm, confirming the results obtained by the Malvern Zetasizer.

#### Robustness to dilution and thermodynamic stability 

The microemulsion is a thermodynamically stable system compared to other types of emulsions. This may be due to the kinetic stability of the system and thus they will not undergo phase separation under stressful conditions.^53^ The selected formula (F7) showed good thermal stability and no phase separation, cracking, or creaming when subjected to excessive dilution with deionized water, centrifugation and freeze-thaw cycles.

### In vivo skin tolerability assessment 

Irritant skin is a common inflammatory skin condition resulted from a single or repetitive exposure to natural products or chemical agents with irritant properties.^54^ Topically, the effect of pure limonene oil and limonene based microemulsion system on the skin of mice over seven consecutive days was studied.

According to the Draize scale, physical appearance of the mice ears showed slight irritant effect with no significant difference (*P* > 0 0.05) as summarized in Table 3.

**Table 3:** Irritation of the mice ears by pure limonene oil and limonene based microemulsion

**Formula**	**Erythema formation score after 24 h**	**Erythema formation score at day 7** ^a^	**Primary skin irritant index**
Pure limonene oil	0.00±0.00	1.70±1.53	Slight irritant
Limonene-based microemulsion (F7)	0.00±0.00	1.30±0.58	Slight irritant

^a^ Values are presented as mean ± SD; (*n* = 3). Data were analyzed using *t*test; (*P* = 0.74).

**Figure 5:**
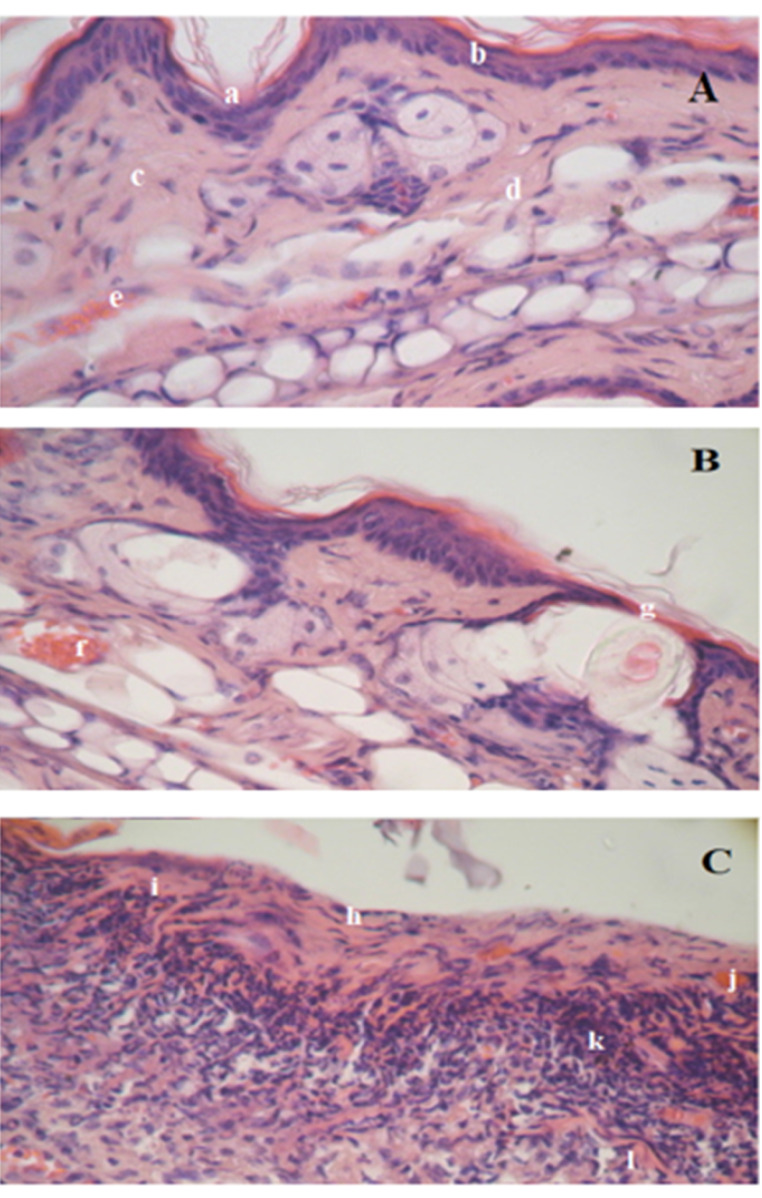
Histological features of the mouse ear (H&E stain). Negative control group [A] shows (a) normal stratum corneum within the epidermis layer, (b) regular basal layer, (c) normal collagen fiber within the dermis layer, (d) normal structure and thickness of dermis layer with regular shape, (e) normal blood flow. Mouse skin treated with limonene-based microemulsion [B] shows (f) loose of epidermis layer and (g) slight hyperanemia within the dermis. Mouse skin treated with D-limonene oil [C] shows (h) hyperkeratosis with hyperplasia, (i) slight spongiosis, (j) slight hyperaemia, (k) Loose perivascular and diffuse infiltrate and (l) fractured collagen.

Nevertheless, for further in-deep assessment of these findings, a histopathological study was carried out on day 7, to spot any irregularity, erythema, edema, or any damage of the underlying tissues. Microscopic observations of histopathologic samples are represented in Figure 5 followed by the mean histological score in Table 4. The samples were scored as described previously by Lashmar et al.^55^ The histological score was obtained by adding the sum of the scores for the epidermal features, hyperaemia, and the dermis features according to the equation proposed by Sintov et al.^56^ The results provided a mean of comparing the inflammatory effects of pure and formulated limonene oil. The formula which scored from 0 to 10 were regarded as not causing any reactions in the mouse skin. Preparations that scored from 11 to 20 caused skin reactions, and those above 21 were considered to cause severe damage to the skin.

**Table 4:** Frequency of histological changes induced by topical application of D-limonene and Microemulsion formulation

	**Control (n = 3)**	**Microemulsion** **(n = 3)**	**D-limonene (n = 3)**
A. Epidermal thickening	0	0	7
B. Increase in cell layers of the stratum granulosum	0	0	5
Hyperkeratosis^a^	0	3	7
Spongiosis^a^	0	0	3
E. Destruction of the epidermis Superficial	0	0	0
Hyperaemia^a^	0	5	5
G. Fractured collagen	0	0	3
H. Infiltration of the dermis	0	0	12
Mean histological score	0	8	42

^a^ Hyperkeratosis; thickening of the stratum corneum, spongiosis; intercellular edema in the epidermis, Hyperaemia; increase in the quantity of blood flow.

As displayed in Figure 5, skin treated with pure D-limonene oil showed compact hyper keratinized stratum corneum with ulcerative eruptions, acanthosis, severe hyperplasia, slight spongiosis, and hyperaemia within the epidermis layer as compared to the negative control skin. The collagen bundles in the dermis layer appeared slightly fractured. In addition to severe infiltration in the upper layer of the dermis was observed, where the number of the cells was more than double as compared to the negative control skin. The average histological score of this group was 42 (> 21) indicating that D-limonene oil caused severe damage to the skin when applied undiluted and directly to the skin. On the contrary, incorporated D-limonene in the microemulsion system was very similar to the untreated skin (negative control) except for slight loose in a small part of the hyperkeratine layer which revealed also slight hyperaemia. The mean score of the mice group treated with the ME system was 8 revealing that no reaction has taken place in the treated area. These results are discrepant to that of Draize score which relied only on visual estimation. Microscopical examination of the histopathological samples is more accurate and indisputable, revealing that the microemulsion system can protect the irritant effect of D-limonene and can be applied safely on the skin. On the same line, eucalyptus oil which is irritant topically,^57^ became not irritant to the skin and showed no histological irregularities when applied on the dorsal side of the rats in form of nanoemulsion according to Sugumar et al^58^

### Stability study 

The main aim of the stability test is to evaluate how the properties of drug products change with time under the impact of different environmental conditions. In this context, the stability of the optimized ME (F7) was evaluated based on its visual appearance, droplet size, PDI, and zeta potential variations through three months of storage period.

**Figure 6:**
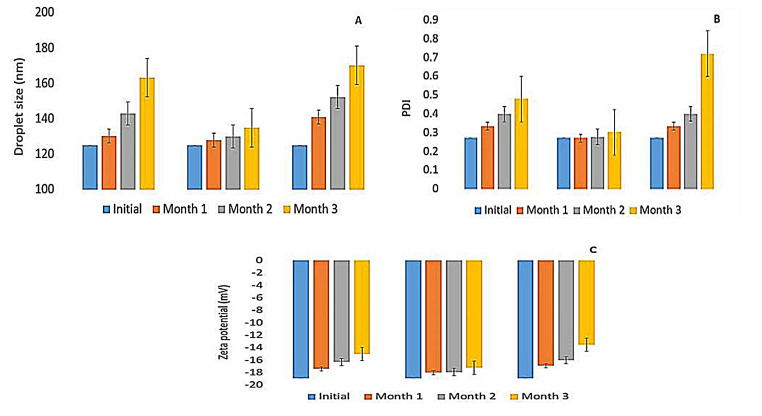
**(A)** droplet size (nm), **(B)** polydispersity index and **(C)** zeta potential (mV) of limonene-based microemulsion (F7) after three months’ storage at 25±2°C, 5±2°C and 0±2°C.

At the end of the storage period, the optimized microemulsion at different temperatures didn’t exhibit any alteration in its visual appearance, with no flocculation, precipitation, or phase separation. At 5±2°C, F7 did not show any significant change in its droplet size, PDI, and its zeta potential during the storage period, due to the presence of a high concentration of Labrasol^®^ that stabilizes the system against Ostwald ripening^59^ and prevents globule self-coalescence.^28,59^ This result is in agreement with those of Okur et al^60^ where hydroquinone-loaded microemulsion showed good stability at 4±1℃ when stored for three months. Nevertheless, at 25±2°C storage of limonene-based microemulsion caused the droplet size and PDI increase significantly from 125±0.123 nm and 0.272±0.009 to 172.88±32.51 nm and 0.661±0.03, respectively (*P*< 0.05) as shown in Figure 6 (A and B) . This change is maybe due to the increase in the ripening rate at a higher temperature that favored the movement of the dispersed particles, particularly the adhesion, collision, and the growth of the droplets in the ME system, via the continuous phase. In addition to a change in the solubility of the non-ionic surfactant in the bulk phase, producing an alteration in the film compressibility.^61^

At 0±2°C, freshly F7 showed good stability as shown previously in thaw/freeze cycles, yet storage for three months altered its size, PDI and zeta (Figure 6), and this could be explained by the presence of the hydrogen bonds between water molecules which upon freezing becomes stronger and cause the matter to expand. This volume expansion leads to coalescence and partial coalescence through the collision between undercooled and frozen droplets. Subsequently, coalesced droplets diffuse into larger particles due to interfacial area reduction in the freezing process, resulting in demulsification of the system.^62^

## Conclusion

In the current study, Gelucire^®^ and limonene-based microemulsion were developed using the spontaneous emulsification method. The optimal formula had unique characteristics, namely; small droplet size (125±0.123 nm), unimodal size distribution (PDI 0.272±0.009), efficient zeta potential (-18.9±2.79 mV) and high percentage transmittance (96.5±0.88 %) with a pH of 6.3. The formulated microemulsion showed good stability against different dilutions and centrifugation. No irritation was observed when apply limonene-based microemulsion on the mice ears as revealed by the histopathological examination. Besides, the formula was stable at 5±2°C with no sign of precipitation or separation for three months. These results suggested that microemulsion is suitable to be used as a prospective aid for the essential oil to minimize its volatility, enhance its stability, mask its dermal irritant effect and maximize its therapeutic efficiency and patient acceptability.

## Ethical Issues

The procedure on animals was performed according to the laws and guidelines provided by the Institutional Animal Care and Use Guidelines (IACUG) at Beirut Arab University, validated by the Ministry of Public Health of an Institutional Review Board (IRB) number: 2020-0045-P-M-93.

## Conflict of Interests

None.
